# Association between electrocardiographic age and cognitive function: findings from the UK biobank and Framingham Heart Study

**DOI:** 10.1093/ehjdh/ztag034

**Published:** 2026-02-18

**Authors:** Bernard Ofosuhene, Huitong Ding, Heaven Y Tatere, Trevor W Vigeant, Shensheng Li, Alexander Bennett, Honghuang Lin

**Affiliations:** Department of Medicine, University of Massachusetts Chan Medical School, 55 Lake Ave N, Worcester, MA 01655, USA; Department of Anatomy and Neurobiology, Boston University Chobanian & Avedisian School of Medicine, Boston, MA, USA; The Framingham Heart Study, Boston University Chobanian & Avedisian School of Medicine, Boston, MA, USA; Department of Medicine, University of Massachusetts Chan Medical School, 55 Lake Ave N, Worcester, MA 01655, USA; Department of Medicine, University of Massachusetts Chan Medical School, 55 Lake Ave N, Worcester, MA 01655, USA; Department of Biology, University of Massachusetts Amherst, Amherst, MA, USA; Department of Medicine, University of Massachusetts Chan Medical School, 55 Lake Ave N, Worcester, MA 01655, USA; Department of Medicine, University of Massachusetts Chan Medical School, 55 Lake Ave N, Worcester, MA 01655, USA

**Keywords:** Deep learning, Electrocardiographic age, Cognitive function

## Abstract

**Aims:**

Biological age derived from 12-lead electrocardiograms (ECGs) using deep learning has emerged as a promising marker of physiological ageing. However, its relationship with cognitive performance remains poorly understood. To investigate the association between ECG-derived ageing and cognitive performance in two large population-based cohorts.

**Methods and results:**

We analysed data from the UK Biobank (UKB) and the Framingham Heart Study (FHS). A deep learning model estimated ECG-based biological age (ECG-age) from ECG waveforms. We calculated the difference between ECG-age and chronological age (Δage), which was used to classify participants into ageing groups: accelerated ageing, normal ageing, and decelerated ageing. Cognitive performance was measured with standardized neuropsychological tests, which were grouped into six cognitive domains and a global cognitive score. Multivariable linear regression models were used to examine the associations of Δage and ageing groups with cognitive performance. Among 59 213 UKB participants (mean age 64.7 ± 7.8 years; 51.7% women) and 6534 FHS participants (mean age 59.5 ± 14.5 years; 55.7% women), the mean absolute error between ECG-age and chronological age was 4.7 and 7.5 years, respectively. In both cohorts, higher Δage was associated with lower global cognitive performance (UKB: β = −0.02, 95% CI: −0.03, −0.02; FHS: β = −0.04, 95% CI: −0.06, −0.02) and poorer performance across multiple cognitive domains.

**Conclusion:**

Electrocardiogram-derived age acceleration is associated with poorer cognitive performance across two independent cohorts. Electrocardiogram-based ageing metrics may serve as scalable, low-cost digital markers to identify individuals at elevated risk for cognitive decline.

## Graphical Abstract

**Graphical Abstract:**
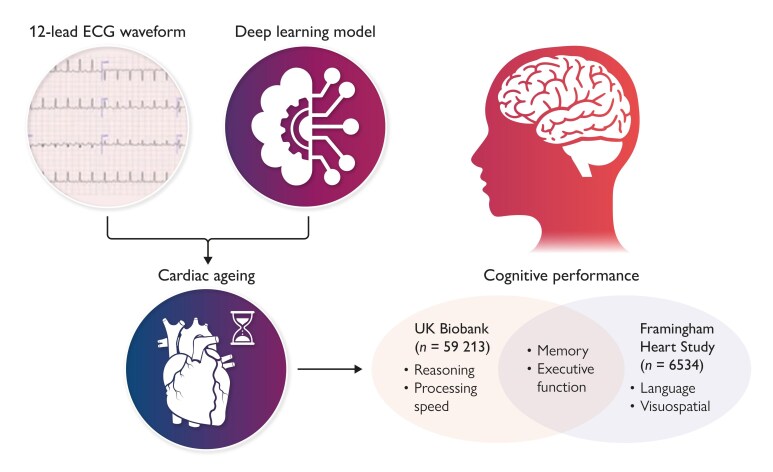

## Introduction

As populations age worldwide, the burden of cognitive impairment and dementia is increasing. This trend has major public health and socioeconomic consequences.^1^ Chronological age is the strongest risk factor for cognitive decline. However, individuals of the same age show wide variation in cognitive function. This variation highlights the limitations of chronological age as a proxy for the biological processes that drive brain ageing.

Biological age reflects the physiological state of an individual’s organ systems. It is increasingly recognized as a more informative indicator of ageing-related outcomes than chronological age, including cognitive decline. Recent advances in artificial intelligence (AI) now allow biological age to be estimated from common clinical data, such as the 12-lead electrocardiogram (ECG).^2^ Deep learning models trained on large ECG datasets can predict an individual’s ‘ECG-age’, which serves as a surrogate marker of cardiovascular ageing. The difference between ECG-age and chronological age, termed delta age (Δage), has been associated with all-cause mortality and cardiovascular disease.^3,4^ Furthermore, cerebrovascular pathology plays a critical role in Alzheimer’s disease (AD), and up to 50% of patients with AD show cerebrovascular abnormalities in addition to AD pathology.^5^ However, it remains largely unknown whether ECG-derived Δage reflects or captures broader systemic ageing processes that are relevant to cognitive function.

Given the close link between cardiovascular and cognitive health,^6^ AI-derived ECG-age may reflect hidden physiological changes that occur before or accompany neurodegeneration. Age-related cerebrovascular dysfunction, including white matter hyperintensities, small vessel disease, and hypoperfusion, contribute to cognitive decline and often co-occur with AD pathology. Therefore, ECG-derived biological ageing may serve as a non-invasive and scalable marker for cognitive ageing, with potential use in early risk stratification.

In this study, we examined the association between ECG-derived biological age and cognitive performance in two cohorts: The UK Biobank (UKB) and the Framingham Heart Study (FHS). We also tested whether individuals with accelerated ECG ageing have poorer global and domain-specific cognitive function. Finally, we aimed to evaluate ECG-derived ageing as a potential digital phenotype for cognitive health in middle-aged and older adults.

## Methods

### Study population

The UKB is a large cohort study that enrolled 502 505 individuals living in the United Kingdom between 2006 and 2010.^7,8^ It collected extensive genetic and phenotypic data from each participant.

The FHS is a long-term community based cohort study that began in 1948.^9^ The Original Cohort included 5209 men and women aged 30–62 years from Framingham, Massachusetts. In 1971, the Offspring Cohort started and enrolled 5124 adult children of the Original Cohort and their spouses. Beginning in 2002, the Third Generation Cohort was recruited and included 4095 children of the Offspring Cohort. To improve diversity, two Omni cohorts were added. Omni 1 was initiated in 1994 and Omni 2 in 2003. These two cohorts enrolled participants from underrepresented racial and ethnic groups. In 2003, the New Offspring Spouse Cohort was also introduced, which includes newly enrolled spouses of Offspring participants. All cohorts have been followed with detailed clinical examinations approximately every 2 to 4 years. These examinations include ECGs, neuropsychological tests, and other health assessments.^10^ For the present analysis, we included FHS participants who had both ECG data and cognitive assessments obtained within 3 years (*n* = 6534).

### Electrocardiogram collection

At UKB imaging assessment centre, resting 12-lead ECGs were collected using a standardized protocol. Recordings were taken both before and during a submaximal cycling exercise. Participants lay on the same examination couch that was used for carotid ultrasound. Electrocardiograms were acquired with the GE Cardiosoft system integrated into the workstation setup. Raw ECG data were extracted from extensible mark up language (XML) files (Data-Field 20205). Each ECG was recorded for 10 s at a sampling rate of 500 Hz. The signals were then re-sampled to 400 Hz and zero-padded to a fixed length of 12 × 4096 samples for input into the deep learning model.

In FHS, ECGs were collected from all participants as part of routine clinical visits. Digital ECG recording began in 1986 using systems including the Marquette MAC/PC and later the MAC 5000 (General Electric). Currently, ECG data are managed and analysed with the MUSE 8 ECG Management System (General Electric). It provides standardized processing of all digitally recorded ECGs.^11^

### Electrocardiogram-age estimated by deep learning

A previously developed deep learning model was used to estimate ECG-age.^12^ This model was originally trained on ECG data from 1 558 415 individuals in the CODE study, a large initiative within the Telehealth Network of Minas Gerais, Brazil.^13^ The CODE dataset includes ECGs recorded in Brazilian primary care settings between 2010 and 2017.^14^ It is one of the most extensive ECG resources available for AI research.^13^ The model architecture is based on a convolutional neural network designed for one-dimensional ECG signals. It consists of five residual blocks. Each block contains two convolutional layers. Comprehensive methodological details have been described in the original publication.^12^ This algorithm was further refined in the FHS, where an accelerated ECG ageing was associated with an increased risk of all-cause mortality and incident cardiovascular disease events.^15^ The Δage was defined as the difference between ECG-age and the individual’s chronological age.

### Electrocardiogram ageing group definition

Participants were classified into three ageing groups consistent with prior studies.^12,15^ The mean absolute error (MAE) between ECG-age and chronological age was used as the classification threshold. Participants whose ECG-age fell within ± MAE of their chronological age were classified as having normal ageing. Those with ECG-age more than MAE below chronological age were classified as having decelerated ageing, reflecting relatively slower biological ageing. Conversely, participants with ECG-age more than MAE above their chronological age were classified as having accelerated ageing, indicating relatively faster biological ageing.

### Cognitive assessment

In UKB, cognitive performance was assessed using eight neuropsychological tests that measured four domains: memory, executive function, reasoning, and processing speed (see Supplementary material online, *Table S1*). The tests included trail making (TMT), tower rearranging, matrix pattern completion, symbol digit substitution, numeric memory, fluid intelligence, reaction time (RT), and pairs matching.^16^ Details of the UKB cognitive assessments have been reported previously.^17,18^ Missing NP test values were imputed using multiple imputations using the chained equations (MICE) method. We applied natural log transformations to TMT and RT. All NP test scores were then recoded so that higher values indicate better cognitive performance.^19^

At FHS, cognitive performance was assessed using 18 NP tests that covered four cognitive domains including memory, executive function, language, and visuospatial function (see Supplementary material online, *Table S1*). Detailed descriptions of each NP test are available in prior publications.^20–22^ Missing NP test data were handled using MICE method. Trails A, Trails B, and HVOT were transformed using the natural logarithm so that higher values indicated better performance. All NP test scores were standardized to *z*-scores (mean = 0, standard deviation = 1).

### Statistical analyses

To examine the association between Δage and cognitive performance, we used the UKB as the discovery cohort and FHS as the replication cohort. To determine the appropriate functional form, we tested a quadratic term for age in regression models for each cognitive domain. Since none of the quadratic terms were statistically significant (all with *P* > 0.05), we proceeded with linear specifications. Our primary analysis used multivariable linear regression models with the global cognitive *z*-score as the outcome variable and Δage as the predictor, adjusting for chronological age, sex, and education. All continuous variables were standardized (mean = 0, SD = 1). In complementary analyses, we replaced Δage with ECG-derived ageing groups (decelerated, normal, accelerated) as the primary exposure. We next conducted secondary analyses to examine whether associations differed across specific cognitive domains (memory, executive function, reasoning, and processing speed). To evaluate potential effect modification by sex, we included a Δage-by-sex interaction term in the models and additionally fit sex-stratified linear regression models for global and domain-specific cognitive scores, adjusting for age and education in each stratum.

## Results

### Cohort descriptive

As shown in *Table 1*, the UKB cohort included 59 213 participants (mean age 64.7 ± 7.8 years, 51.7% women). The MAE between ECG-age and chronological age was 4.7 ± 3.5 years. Using this MAE, participants were classified into three ageing groups: decelerated (*n* = 12,694, 21.4%), normal (*n* = 33,763, 57.0%), and accelerated ageing (*n* = 12,756, 21.5%). Framingham Heart Study served as the replication cohort and included 6534 participants (mean age 59.5 ± 14.5 years, 55.7% women). The MAE between ECG-age and chronological age was higher (7.5 ± 5.9 years), but the distribution of ageing groups was similar: decelerated (*n* = 1,875, 28.7%), normal (*n* = 3,843, 58.8%), and accelerated ageing (*n* = 816, 12.5%).

**Table 1:** Characteristics of the study participants

Characteristics	UKB (*n* = 59 213)			FHS (*n* = 6534)		
	Decelerated ageing (*n* = 12 694)	Normal ageing (*n* = 33 763)	Accelerated ageing (*n* = 12 756)	Decelerated ageing (*n* = 1875)	Normal ageing (*n* = 3843)	Accelerated ageing (*n* = 816)
Age, years	65.0 ± 7.6	64.5 ± 7.9	65.0 ± 7.8	68.1 ± 12.5	57.3 ± 13.5	50.7 ± 13.8
Women	7758 (61.1%)	17 658 (52.3%)	5196 (40.7%)	1060 (56.5%)	2122 (55.2%)	456 (55.9%)
Education						
Low	2084 (16.4%)	5900 (17.5%)	2402 (18.8%)	743 (39.7%)	1093 (28.5%)	212 (26.0%)
Intermediate	3815 (30.1%)	10 914 (32.3%)	4199 (32.9%)	451 (24.1%)	1077 (28.1%)	227 (27.8%)
High	6795 (53.5%)	16 949 (50.2%)	6155 (48.3%)	676 (36.1%)	1664 (43.4%)	377 (46.2%)
BMI, kg/m^2^	25.7 ± 4.1	26.6 ± 4.4	27.4 ± 4.8	26.8 ± 4.5	27.9 ± 5.4	28.5 ± 6.4
SBP, mmHg	139 ± 20	143 ± 20	148 ± 21	129 ± 20	125 ± 19	125 ± 19
DBP, mmHg	77 ± 10	79 ± 11	82 ± 11	72 ± 10	75 ± 10	76 ± 10

Categorical variables were represented as *n* (%), whereas continuous variables were represented as mean ± standard deviation (SD).

In the UKB, education was categorized as follows: high (college or university degree), intermediate (A/AS levels or equivalent, O levels/GCSEs or equivalent), and low (none of the aforementioned). In the FHS, education was categorized as follows: high (college graduate), intermediate (some college), low (high school did not graduate, high school graduate).

### Association between electrocardiogram age and cognitive performance

*Table 2* shows consistent associations between higher Δage and poorer cognitive performance. In UKB, higher Δage was associated with lower global cognition, memory, executive function, reasoning, and processing speed (all *P* < 0.01). In FHS, higher Δage was associated with lower global cognition, memory, executive function, language, and visuospatial function (all *P* < 0.01). Effect sizes were generally larger in FHS than in UKB.

**Table 2:** Association of Δage with cognitive performance

Cognitive domain	UKB			FHS		
	β	95% CI	*P*	β	95% CI	*P*
**Global cognitive function**	−0.02	−0.03, −0.02	**<0.001**	−0.04	−0.06, −0.02	**<0.001**
**Memory**	−0.01	−0.02, −0.003	**0.008**	−0.03	−0.05, −0.01	**0.003**
**Executive function**	−0.02	0.02, −0.01	**<0.001**	−0.05	−0.07, −0.03	**<0.001**
**Reasoning**	−0.02	−0.03, −0.02	**<0.001**	—	—	—
**Processing speed**	−0.02	−0.03, −0.02	**<0.001**	—	—	—
**Language**	—	—	—	−0.04	−0.06, −0.01	**0.003**
**Visuospatial**	—	—	—	−0.04	−0.06, −0.02	**<0.001**

***P*** **-values highlighted in bold are statistically significant.**

Building on the overall associations of Δage with cognition, we next examined whether these relationships differed by sex (see Supplementary material online, *Tables S2* and *S3*). In UKB, higher Δage was associated with poorer global and domain-specific performance in both men and women. The effect sizes were generally larger in men compared with women. There are modest sex differences in global cognition and executive function (interaction *P* < 0.05). In contrast, sex differences were less apparent in FHS. Higher Δage was associated with lower global cognition and poorer performance across global cognition, executive function, and visuospatial function in both men and women. Effect sizes were similar by sex for global cognition, executive function, and visuospatial function, and slightly larger in women for language. But, there is no strong evidence that associations differed by sex in this cohort (interaction *P* > 0.05).

To complement the continuous Δage analyses, we next examined ECG-derived ageing groups (accelerated, normal, decelerated) in relation to cognitive performance (*Tables 3* and *4*). Compared with normal ageing, accelerated ageing was associated with lower global cognitive function in both cohorts (UKB: β = −0.02, 95% CI: −0.04, −0.004; FHS: β = −0.12, 95% CI: −0.17, −0.06). In contrast, decelerated ageing was associated with better global cognition in UKB (β = 0.03, 95% CI: 0.01, 0.05), with a similar but borderline association in FHS (β = 0.04, 95% CI: −0.002, 0.09; *P* = 0.06). We then evaluated domain-specific performance. In UKB, accelerated ageing was linked to poorer reasoning (β = −0.02, 95% CI: −0.04, −0.001) and processing speed (β = −0.03, 95% CI: −0.05, −0.01). The associations with memory and executive function were negative but not statistically significant. Decelerated ageing in UKB was associated with better executive function (β = 0.02, 95% CI: 0.002, 0.04), reasoning (β = 0.04, 95% CI: 0.02, 0.06), and processing speed (β = 0.03, 95% CI: 0.01, 0.05). In FHS, accelerated ageing showed consistently worse performance across all domains, including memory (β = −0.08, 95% CI: −0.14, −0.02), executive function (β = −0.12, 95% CI: −0.18, −0.06), language (β = −0.08, 95% CI: −0.15, −0.01), and visuospatial function (β = −0.12, 95% CI: −0.18, −0.06). Decelerated ageing was associated with better executive function (β = 0.05, 95% CI: 0.01, 0.10) and language (β = 0.06, 95% CI: 0.01, 0.11). Associations with memory and visuospatial function were positive but not statistically significant.

**Table 3:** Association of accelerated ageing with cognitive performance

Cognitive domain	UKB			FHS		
	β	95% CI	*P*	β	95% CI	*P*
**Global cognitive function**	−0.02	−0.04, −0.004	**0.02**	−0.12	−0.17, −0.06	**<0.001**
**Memory**	−0.01	−0.03, 0.01	0.35	−0.08	−0.14, −0.02	**0.007**
**Executive function**	−0.01	−0.03, 0.01	0.23	−0.12	−0.18, −0.06	**<0.001**
**Reasoning**	−0.02	−0.04, −0.001	**0.04**	—	—	—
**Processing speed**	−0.03	−0.05, −0.01	**0.002**	—	—	—
**Language**	—	—	—	−0.08	−0.15, −0.01	**0.02**
**Visuospatial**	—	—	—	−0.12	−0.18, −0.06	**<0.001**

***P*** **-values highlighted in bold are statistically significant.**

**Table 4:** Association of decelerated aging with cognitive performance

Cognitive domain	UKB			FHS		
	β	95% CI	*P*	β	95% CI	*P*
**Global cognitive function**	0.03	0.01, 0.05	**0.002**	0.04	−0.002, 0.09	0.06
**Memory**	0.003	−0.02, 0.02	0.75	0.03	−0.02, 0.07	0.25
**Executive function**	0.02	0.002, 0.04	**0.047**	0.05	0.01, 0.10	**0.02**
**Reasoning**	0.04	0.02, 0.06	**<0.001**	—	—	—
**Processing speed**	0.03	0.01, 0.05	**<0.001**	—	—	—
**Language**	—	—	—	0.06	0.01, 0.11	**0.03**
**Visuospatial**	—	—	—	0.01	−0.03, 0.06	0.57

***P*** **-values highlighted in bold are statistically significant.**

## Discussion

In this large, population-based study leveraging data from both the UKB and the FHS, we found that an accelerated ECG ageing was significantly associated with poorer cognitive performance. Associations were observed for both global and domain-specific scores, and the overall pattern was consistent across two independent cohorts.

Electrocardiogram-age estimated from deep-learning models applied to 12-lead ECGs may therefore serve as a novel, scalable digital biomarker of cognitive ageing. In UKB, greater Δage related to lower global cognition, memory, executive function, reasoning, and processing speed. Comparable associations were observed in FHS. The cross-cohort consistency supports the generalizability of ECG-age as an ageing index. The inverse relationship between Δage and cognitive performance is consistent in men and women, with modest sex-specific variation in UKB but largely comparable effects in FHS. Biologically, ECG-based ageing may capture cumulative cardiovascular and autonomic dysregulation-processes implicated in cognitive decline. That a short, resting ECG can correlate with cognition underscores the possibility that systemic physiological ageing leaves a measurable electrophysiologic signature.

This study has several notable strengths, including two large, well-characterized population-based cohorts with waveform-level ECG data and standardized neuropsychological assessments; the application of a previously validated deep learning model trained on more than 1.5 million ECGs; and a harmonized definition of Δage that enhances interpretability across diverse populations.

Nonetheless, several limitations should be acknowledged. First, the cross-sectional design of this study limits causal inference. Longitudinal follow-up is needed to determine whether Δage predicts later cognitive decline. In addition, any future use of Δage for risk stratification or early detection would require formal prognostic modelling and prospective validation. Second, differences in ECG acquisition equipment and signal-processing pipelines may introduce measurement heterogeneity across cohorts. Third, the UKB and FHS used different NP tests. In UKB, processing speed showed the strongest and most consistent associations with ageing groups, consistent with a previous study.^6^ However, the NP tests in processing speed used in UKB were not available in FHS. Framingham Heart Study also had a smaller sample size and differed from UKB in population health and life expectancy. These differences limit direct comparison of domain-specific associations, even though ECG-age and Δage were defined in an age-anchored and cohort-specific way. Finally, although models were adjusted for age, sex, and education, residual confounding by unmeasured comorbidities, lifestyle factors, socioeconomic status, or genetic susceptibility cannot be ruled out. Future research should evaluate the longitudinal prognostic value of ECG-derived Δage, evaluate whether it provides incremental predictive value beyond established risk factors, and explore its integration with other digital and biological ageing biomarkers to improve dementia risk stratification and early detection.

## Conclusion

In summary, ECG-derived biological ageing is associated with cognitive performance in middle-aged and older adults. These findings support ECG-age as a scalable, non-invasive, accessible marker for identifying individuals at elevated risk for cognitive decline. Future work should test whether ECG-age acceleration predicts longitudinal cognitive trajectories and assess its incremental prognostic value when integrated with other digital and biological ageing markers to improve dementia risk stratification.

## Ethics statement

The UKB operates under a generic research tissue bank approval from the NHS North West Research Ethics Committee (21/NW/0157). It also covers this study. Therefore, no separate ethics application was needed. This study used UKB data accessed under application ID 76269. The study procedures at FHS were approved by the Institutional Review Board of Boston University Medical Campus. All participants provided written informed consent.

## Supplementary Material

ztag034_Supplementary_Data

## Data Availability

Data for this research were sourced from the UK Biobank Resource (Application ID 76269) and the Framingham Heart Study approved by the Institutional Review Board of Boston University Medical Campus. Interested researchers can access these datasets upon registration and application via these institutions’ official portal. For further details, please contact the corresponding author.

## References

[1] Nichols E, Steinmetz JD, Vollset SE, Fukutaki K, Chalek J, Abd-Allah F, et al Estimation of the global prevalence of dementia in 2019 and forecasted prevalence in 2050: an analysis for the global burden of disease study 2019. The Lancet Public Health 2022;7:e105–e125. 34998485 10.1016/S2468-2667(21)00249-8 PMC8810394

[2] van der Wall HE, Hassing G-J, Doll R-J, van Westen GJ, Cohen AF, Selder JL, et al Cardiac age detected by machine learning applied to the surface ECG of healthy subjects: creation of a benchmark. J Electrocardiol 2022;72:49–55. 35306294 10.1016/j.jelectrocard.2022.03.001

[3] Ladejobi AO, Medina-Inojosa JR, Shelly Cohen M, Attia ZI, Scott CG, LeBrasseur NK, et al The 12-lead electrocardiogram as a biomarker of biological age. Eur Heart J Digit Health 2021;2:379–389. 36713596 10.1093/ehjdh/ztab043 PMC9707884

[4] Attia ZI, Friedman PA, Noseworthy PA, Lopez-Jimenez F, Ladewig DJ, Satam G, et al Age and sex estimation using artificial intelligence from standard 12-lead ECGs. Circ Arrhythm Electrophysiol 2019;12:e007284. 31450977 10.1161/CIRCEP.119.007284 PMC7661045

[5] Snyder HM, Corriveau RA, Craft S, Faber JE, Greenberg SM, Knopman D, et al Vascular contributions to cognitive impairment and dementia including Alzheimer's disease. Alzheimers Dement 2015;11:710–717. 25510382 10.1016/j.jalz.2014.10.008 PMC4731036

[6] Iakunchykova O, Schirmer H, Vangberg T, Wang Y, Benavente ED, van Es R, et al Machine-learning-derived heart and brain age are independently associated with cognition. Eur J Neurol 2023;30:2611–2619. 37254942 10.1111/ene.15902

[7] Sudlow C, Gallacher J, Allen N, Beral V, Burton P, Danesh J, et al UK biobank: an open access resource for identifying the causes of a wide range of complex diseases of middle and old age. PLoS Med 2015;12:e1001779. 25826379 10.1371/journal.pmed.1001779 PMC4380465

[8] Bycroft C, Freeman C, Petkova D, Band G, Elliott LT, Sharp K, et al The UK biobank resource with deep phenotyping and genomic data. Nature 2018;562:203–209. 30305743 10.1038/s41586-018-0579-z PMC6786975

[9] Tsao CW, Vasan RS. Cohort profile: the framingham heart study (FHS): overview of milestones in cardiovascular epidemiology. Int J Epidemiol 2015;44:1800–1813. 26705418 10.1093/ije/dyv337 PMC5156338

[10] Andersson C, Johnson AD, Benjamin EJ, Levy D, Vasan RS. 70-year legacy of the framingham heart study. Nat Rev Cardiol 2019;16:687–698. 31065045 10.1038/s41569-019-0202-5

[11] Magnani JW, Newton-Cheh C, O'Donnell CJ, Levy D. Development and application of a longitudinal electrocardiogram repository: the framingham heart study. J Electrocardiol 2012;45:673–676. 22832152 10.1016/j.jelectrocard.2012.06.016 PMC3483375

[12] Lima EM, Ribeiro AH, Paixão GM, Ribeiro MH, Pinto-Filho MM, Gomes PR, et al Deep neural network-estimated electrocardiographic age as a mortality predictor. Nat Commun 2021;12:5117. 34433816 10.1038/s41467-021-25351-7 PMC8387361

[13] Siontis KC, Noseworthy PA, Attia ZI, Friedman PA. Artificial intelligence-enhanced electrocardiography in cardiovascular disease management. Nat Rev Cardiol 2021;18:465–478. 33526938 10.1038/s41569-020-00503-2 PMC7848866

[14] Ribeiro ALP, Paixao GM, Gomes PR, Ribeiro MH, Ribeiro AH, Canazart JA, et al Tele-electrocardiography and bigdata: the CODE (clinical outcomes in digital electrocardiography) study. J Electrocardiol 2019;57:S75–S78. 10.1016/j.jelectrocard.2019.09.008 31526573

[15] Brant LC, Ribeiro AH, Pinto-Filho MM, Kornej J, Preis SR, Fetterman JL, et al Association between electrocardiographic age and cardiovascular events in community settings: the framingham heart study. Circ Cardiovasc Qual Outcomes 2023;16:e009821. 37381910 10.1161/CIRCOUTCOMES.122.009821 PMC10524985

[16] Lyall DM, Cullen B, Allerhand M, Smith DJ, Mackay D, Evans J, et al Cognitive test scores in UK biobank: data reduction in 480,416 participants and longitudinal stability in 20,346 participants. PLoS One 2016;11:e0154222. 27110937 10.1371/journal.pone.0154222 PMC4844168

[17] Zimmerman SC, Brenowitz WD, Calmasini C, Ackley SF, Graff RE, Asiimwe SB, et al Association of genetic variants linked to late-onset Alzheimer disease with cognitive test performance by midlife. JAMA Netw Open 2022;5:e225491. 35377426 10.1001/jamanetworkopen.2022.5491 PMC8980909

[18] Fawns-Ritchie C, Deary IJ. Reliability and validity of the UK biobank cognitive tests. PLoS One 2020;15:e0231627. 32310977 10.1371/journal.pone.0231627 PMC7170235

[19] Wang B, Chibnik LB, Choi SH, Blacker D, DeStefano AL, Lin H. Association of genetic risk of Alzheimer’s disease and cognitive function in two European populations. Sci Rep 2025;15:6410. 39984543 10.1038/s41598-025-90277-9 PMC11845681

[20] Ding H, Wang B, Hamel AP, Karjadi C, Ang TF, Au R, et al Exploring cognitive progression subtypes in the framingham heart study. Alzheimers Dement 2024;16:e12574. 10.1002/dad2.12574 PMC10955221 38515438

[21] Au R, Seshadri S, Wolf PA, Elias MF, Elias PK, Sullivan L, et al New norms for a new generation: cognitive performance in the framingham offspring cohort. Exp Aging Res 2004;30:333–358. 15371099 10.1080/03610730490484380

[22] Shishtar E, Rogers GT, Blumberg JB, Au R, Jacques PF. Long-term dietary flavonoid intake and change in cognitive function in the framingham offspring cohort. Public Health Nutr 2020;23:1576–1588. 32090722 10.1017/S136898001900394X PMC7196005

