# Supplementary material for: Association between electrocardiographic age and cognitive function: findings from the UK biobank and Framingham Heart Study
Source: Eur Heart J Digit Health. 2026 Feb 18;7(3):ztag034. doi: 10.1093/ehjdh/ztag034 (PMC12966501; doi:10.1093/ehjdh/ztag034)
Supplement: ztag034_Supplementary_Data [file ztag034_supplementary_data.docx]

**Supplemental Table 1.** Cognitive domains and corresponding NP tests in UKB and FHS.

| **Cognitive domain** | **NP test** | |
| --- | --- | --- |
|  | **UKB** | **FHS** |
| Memory | Numeric memory (MEMN)  Pairs matching (MEMP) | WMS-III Logical Memory–Immediate Recall  WMS-III Logical Memory–Delayed Recall  WMS-III Logical Memory–Delayed Recognition  WMS-III Visual Reproductions Immediate Recall  WMS-III Visual Reproductions Delayed Recall  WMS-III Visual Reproductions Delayed Recognition  WMS-III Paired Associates Immediate Recall  WMS-III Paired Associates–Delayed Recall |
| Executive function | Trail making B (TMT B)  Tower rearranging (TWR) | Trail Making Test A (Trails A) and Test B (Trails B)  WAIS - Digit Span Forward & Backward  WAIS - Similarities |
| Reasoning | Fluid intelligence (FI)  Matrix pattern completion (MAT) | -- |
| Processing speed | Symbol digit substitution (SDS)  Reaction time (RT) | -- |
| Language | -- | Controlled Oral Word Association Test (FAS)  Boston Naming Test – 36-item version  Boston Naming Test – 30-item version  Boston Naming Test – 10-item version |
| Visuospatial | -- | Hooper Visual Organization Test |

**Supplemental Table 2**. Sex-stratified associations of Δage with global and domain-specific cognitive performance in UKB

| **Cognitive domain** | **Male** | | | **Female** | | | **Interaction *P* value** |
| --- | --- | --- | --- | --- | --- | --- | --- |
|  | **β** | **95% CI** | ***P*** | **β** | **95% CI** | ***P*** |  |
| Global cognition | -0.03 | -0.04, -0.02 | <0.001 | -0.01 | -0.02, -0.003 | 0.02 | 0.01 |
| Memory | -0.02 | -0.03, -0.01 | 0.003 | -0.004 | -0.01, 0.01 | 0.48 | 0.09 |
| Executive function | -0.03 | -0.04, -0.01 | <0.001 | -0.01 | -0.02, 0.01 | 0.34 | 0.01 |
| Reasoning | -0.03 | -0.04, -0.02 | <0.001 | -0.01 | -0.02, -0.002 | 0.02 | 0.02 |
| Processing speed | -0.03 | -0.04, -0.02 | <0.001 | -0.02 | -0.03, -0.01 | <0.001 | 0.08 |

**Supplemental Table 3**. Sex-stratified associations of Δage with global and domain-specific cognitive performance in FHS

| **Cognitive domain** | **Male** | | | **Female** | | | **Interaction *P* value** |
| --- | --- | --- | --- | --- | --- | --- | --- |
|  | **β** | **95% CI** | ***P*** | **β** | **95% CI** | ***P*** |  |
| Global cognition | -0.04 | -0.07, -0.01 | 0.005 | -0.04 | -0.07, -0.02 | 0.001 | 0.34 |
| Memory | -0.03 | -0.06, 0.001 | 0.06 | -0.03 | -0.06, -0.004 | 0.03 | 0.28 |
| Executive function | -0.05 | -0.08, -0.02 | 0.002 | -0.05 | -0.07, -0.02 | <0.001 | 0.81 |
| Language | -0.02 | -0.06, 0.01 | 0.19 | -0.04 | -0.08, -0.01 | 0.009 | 0.17 |
| Visuospatial | -0.04 | -0.08, -0.01 | 0.012 | -0.04 | -0.06, -0.01 | 0.01 | 0.75 |
